# Bin-CE: A comprehensive web application to decide upon the best set of outcomes to be combined in a binary composite endpoint

**DOI:** 10.1371/journal.pone.0209000

**Published:** 2018-12-13

**Authors:** Josep Ramon Marsal, Ignacio Ferreira-González, Aida Ribera, Gerard Oristrell, Jose Ignacio Pijoan, David García-Dorado

**Affiliations:** 1 CIBER de Epidemiologia y Salud Pública (CIBERESP), Barcelona, Spain; 2 Epidemiology Unit of the Cardiology Department, University Vall d’Hebron Hospital, Barcelona, Spain; 3 Unitat de Suport a la Recerca Lleida, Institut Universitari de Investigació en Atenció Primària Jordi Gol (IDIAP Jordi Gol), Lleida, Spain; 4 Department of Pediatrics, Obstetrics and Gynecology and Public Health of Univestat Autònoma de Barcelona, Cerdanyola del Vallés, Spain; 5 Department of Medicine of Universitat Autònoma de Barcelona, Cerdanyola del Vallés, Spain; 6 Clinical Epidemiology Unit, BioCruces Health Research Institute, Hospital Universitario Cruces, Bizkaia, Spain; 7 CIBER Cardiovascular (CIBERCV), Barcelona, Spain; University Medical Center Gottingen, GERMANY

## Abstract

The estimation of the Sample Size Requirement (SSR) when using a binary composite endpoint (i.e. two or more outcomes combined in a unique primary endpoint) is not trivial. Besides information about the rate of events for each outcome, information about the strength of association between the outcomes is crucial, since it can determine an increase or decrease of the SSR. Specifically, the greater the strength of association between outcomes the higher the SSR. We present *Bin-CE*, a free tool to assist clinicians for computing the SSR for binary composite endpoints. In a first step, the user enters a set of candidate outcomes, the assumed rate of events for each outcome and the assumed effect of therapy on each outcome. Since the strength of the association between outcomes is usually unknown, a semi-parametric approach linking the a priori clinical knowledge of the potential degree of association between outcomes with the exact values of these parameters was programmed with *Bin-CE*. *Bin-CE* works with a recursive algorithm to choose the best combination of outcomes that minimizes the SSR. In addition, *Bin-CE* computes the sample size using different algorithms and shows different figures plotting the magnitude of the sample size reduction, and the effect of different combinations of outcomes on the rate of the primary endpoint. Finally, *Bin-CE* is programmed to perform sensitivity analyses. This manuscript presents the mathematic bases and introduces the reader to the use of *Bin-CE* using a real example.

## Introduction

The use of a binary Composite Endpoint (CE) in clinical trials, defined as the combination of two or more dichotomous variables in a unique endpoint, is common[1,2]. Particularly when a binary CE is used as the primary endpoint, a patient experiences the primary endpoint if any of the specific components occurs [2]. The two main reasons for using CE instead of a single primary outcome [3–6] are a) the utility of the CE to assess the ‘net benefit’ of a therapy and b) the utility of the CE to reduce the Sample Size Requirement (SSR) by increasing the total number of observed events [7]. For instance, an intervention designed to decrease the rate of myocardial infarction in primary prevention by reducing LDL cholesterol, may reduce total cardiovascular mortality but also the rate of cerebrovascular events. Thus, the use of the CE “Major Adverse Cardiovascular Event (MACE)” including the outcomes myocardial infarction, cardiovascular mortality, and stroke could better capture the net benefit of the intervention. However, this use of CE can also complicate the interpretation of the results of the trial [3,5,6,8–10].

The potential of a binary CE to reduce the SSR is closely related to the magnitude of association between components[11]. In this sense, not all CE have the same potential in the SSR reduction. For example, imagine a hypothetic and bizarre binary CE defined as “Acute Coronary Syndrome (ACS)” or “Troponin Elevation above the normal level”. A patient experiences the CE if any of both events occurs. However, both variables are so strongly associated that they practically mean the same. Therefore, it is unlikely that the combination of them in a binary CE increases the number of events and thus its capacity to reduce SSR. On the other hand, consider a binary CE defined as “ACS” or “stroke”. In this case, although it is anticipated that a certain number of patients may suffer both events these variables are “moderately correlated”, and the number of patients experiencing at least one of the components would be expected to be higher than the number of patients experiencing ACS or stroke if they had been employed as a single primary outcome [12]. In a previous work [12] we assessed the impact of the strength of the association between two components of a binary CE on the SSR. Specifically, the stronger the association between both components the lower the potential reduction of SSR. In addition, the potential impact of the strength of the association between components to modulate the SSR is influenced by both the prevalence of outcomes and the effect of the therapy on each outcome.

In the present work, our previous findings with a binary CE with only two dichotomous components are generalized to a binary CE with k dichotomous components. Specifically, we present an Internet accessible computational tool that incorporates a simple method to assess the SSR and helps a trialist to decide upon combining a set of candidate outcomes in a unique CE. The algorithm has been programmed in a free tool that, starting from *k* possible candidate outcomes, finds the best combination which minimizes the SSR, using or not all k components. This tool named Bin-CE is available as a beta version in https://uesca-apps.shinyapps.io/bincep/. We also present a numeric example to illustrate the inputs and outputs of Bin-CE.

## Material and methods

### Notation and assumptions

We define a *Relevant Endpoint* (RE) as the outcome that is assumed to drive the main effect of the therapy. An *Additional Endpoint* (AE) is another outcome that the researcher considers to combine with the RE in a binary CE to reduce the SSR. A CE could be built up from one RE and *k* additional endpoints. For simplicity, the statistical test applied by default is the asymptotic approximation to the Normal distribution of the difference in the proportion of events between groups[7,13,14]. However, other approximations could be selected for those SSRs that are assumed not be large (i.e. arcsine approximation and with/without correction). We shall consider also for simplicity a RCT with only two treatment arms. The aim of the algorithm is to compute the SSR of both the CE (SSR_CE_) and the RE (SSR_RE_). If the SSR of the CE is smaller than the SSR of the single RE then researcher would prefer the use of the CE [15] instead of the RE alone (i.e. SSR_CE_ < SSR_RE_).

Let *X*_*ijk*_ be the binary response of the *j-th* patient (j = 1, …, n_i_) in the *i-th* group (i = 1,2) for the *k-th* outcome (k = 1, …, K). The algorithm assumes the same number of patients in each group (n_1_ = n_2_) and that at least two outcomes are considered to be combined (K>1). The *Relevant Endpoint* is codified with k = 1 and the *Additional Endpoint* uses the remaining subscripts (k>1). *E*(*X*_*ijk*_) = *π*_*ik*_ and *V*(*X*_*ijk*_) = *π*_*ik*_(1−*π*_*ik*_). Imagine a specific two-component binary CE that combines two outcomes, the RE (*X*_*ijk*_ = 1) and the k’-th AE (*X*_*ijk* = *k*′_), we denote this as $X_{ij}^{1k\prime }$ and note that is equal to 1 whenever *X*_*ijk* = *1*_ *or X*_*ijk* = *k*′_ are equal to 1 and 0 otherwise. $X_{ij}^{1k\prime }$ is distributed as a Bernoulli random variable with mathematical expectation:

$$E(X_{ij}^{1k\prime })=Prob(X_{ij}^{1k\prime }=1)=Prob\left((X_{ij1}=1)\cup (X_{ijk\prime }=1)\right)=Prob(X_{ij1}=1)+Prob(X_{ijk\prime }=1)-Prob\left((X_{ij1}=1)\cap (X_{ijk\prime }=1)\right)=\pi_{i1}+\pi_{ik\prime }-\pi_{i,1k\prime }$$ (1),

where *π*_*i*,1*k*′_ corresponds to the probability of both outcomes (i.e. RE and the k’-th AE) happening together. Note that with these six parameters (*π*_*i* = 1,1_,*π*_*i* = 1,*k*′_,*π*_*i* = 1,1*k*′_,*π*_*i* = 2,1,_*π*_*i* = 2,*k*′_,*π*_*i* = 2,1*k*′_) the SSR of the RE and the CE can be determined[7,16] and thus it can become apparent whether it is worth the combination of the RE with the AE in a CE. Usually *π*_*i*1_,*π*_*ik*_, are known, but *π*_*i*,1*k*′_ is sometimes unknown[17].

It should be noted that the parameter *π*_*i*,1*k*′_ equals the joint probability between the RE and the k’-th AE and it measures the strength of association between them. We assume that the association between outcomes is the same in both study groups. The probability of both outcomes occurring together is the same in the two arms. Henceforth *π*_*i*,1*k*′_ it will be referred as *π*_1*k*′_.

### Quantification of the strength of association between components of a CE

Although there are many different coefficients to quantify the strength of association between components of a binary CE (see Supplementary Material for some examples, S1 File), we have chosen the join probability for simplicity because the other coefficients are more or less complex functions derived from both the join probability and the marginal probabilities.

On the early sixties, the work by Fréchet[18] on the combination of probabilities of events and by Bahadur[19] on the joint probability distribution of binary random variables, characterized the probability of two outcomes happening together (*π*_*kk*′_). It was shown that the distribution is bounded between a lower and an upper limit and that not all values between 0 and 1 are possible. These bounds, known as Fréchet bounds, depend on the marginal prevalence of each outcome as follows:

$$0\leq Low(\pi_{i1},\pi_{ik^{\prime }})=max\{0;\pi_{i1}+\pi_{ik^{\prime }}-1\}\leq \pi_{1k^{\prime }}\leq min\{\pi_{i1},\pi_{ik}\}=Up(\pi_{i1},\pi_{ik\prime })\leq 1$$ (2)

When *π*_1*k*′_ = *π*_*i*1_*π*_*ik*′_ both outcomes are considered as not being associated or as independent events. For instance, imagine a CE including the outcomes “ACS or stroke”, these are considered independent if the probability of a patient experiencing an ‘ACS’ is the same regardless the patient had a ‘stroke’ previously and vice versa.

### Bin-CE algorithm

Bin-CE is a free available web application that can be accessed using a web browser (https://uesca-apps.shinyapps.io/bincep/). Bin-CE has been programmed using the Shiny library on R Studio. The R statistical language[20] has been used to develop Bin-CE App on R Studio. Bin-CE can be directly run by connecting with the Shiny server https://www.shinyapps.io/) without need to install neither R Studio or Shiny package. Nevertheless, users with programming notions of R language can download the source code at Supplementary Material, S2. File All necessary functions to implement Bin-CE can be found in S2 File. Functions, figures and general structures have been programed by ourselves. Using the Supplementary Material 2 you can replicate the examples given in this paper and Supplementary Material 3 contributes with a deeper mathematical approach.

**Fig 1:**
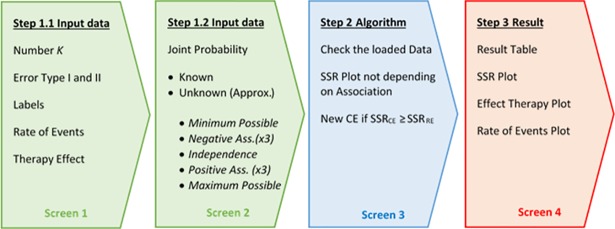
Bin-CE workflow.

Bin-CE has been programmed to select, from a set of candidate binary outcomes (K≤10), the combination that minimizes the SSR. The four-screens that conform Bin-CE are automatically refreshed when any input data changes (Fig 1). The first two screens are used to upload all necessary data. The third screen is used only to check all input data and to present the initial results. When Bin-CE detects any inconsistency in the input data such as a joint probability outside the Fréchet bounds, it is automatically corrected. For example, consider that the user introduces a rate of 6% for the RE and 10% for the AE and a joint probability value of 8%. The joint probability value is not possible since in this case, it is defined only in the range 0% to 6%. Accordingly, Bin-CE automatically corrects this mistake by assigning the product of marginal probabilities for the joint probability (i.e. 0.6%), assuming the non-association scenario. Finally, the last screen displays the best combination of outcomes (if any exists) in a CE as well as the corresponding SSR. This screen also shows the intermediate steps of algorithm iterations.

### Analysis workflow

#### Step 1.1: Input data (Screen 1: Rate of events and effect of the therapy)

**Fig 2:**
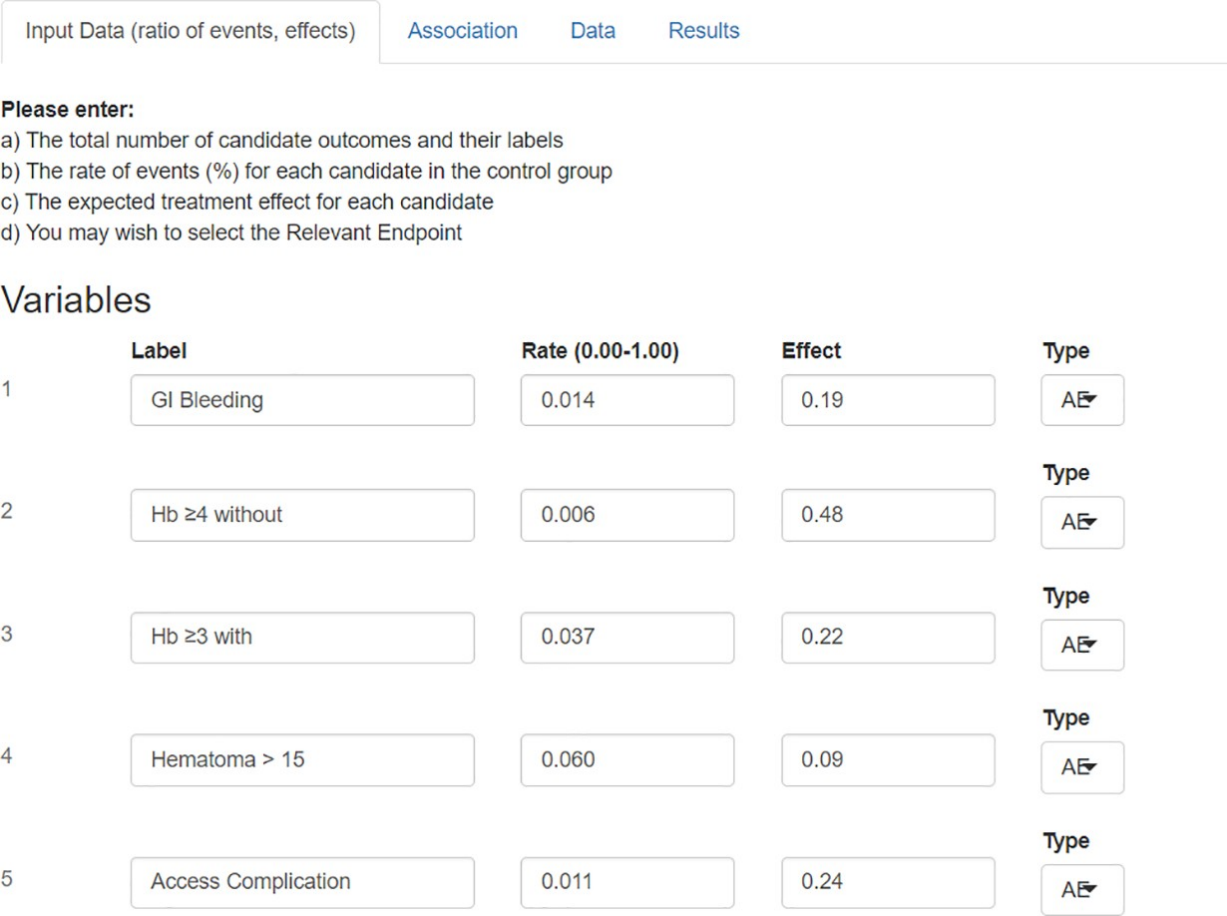
Data showed on screen 1: Rate of events and effect of the therapy. On the first screen (Input Data), the user fixes the number of outcomes, the hypothesis contrast and the type I/II errors assumed. Then the user assigns the following parameters a) the label of each outcome, b) the rate of events in the control group, c) the effect of the therapy measured as a risk ratio and d) if any of the outcomes is considered as the RE.

Bin-CE allows up to nine outcomes that may be combined with the RE. Bin-CE permits allows uploading the label, the rate of events (*π*_*i* = 1,*k*_) in the control group, and the assumed effect of the therapy for each outcome. In addition, the user can choose the Type I and Type II errors. The effect can be assessed using different parameters (*i*.*e*. *difference of proportions*, *rate-ratio*, *odds ratio*). For the following discussion we will assume that the effect is estimated by the Relative Risk (RR) (*π*_*i* = 1,*k*_/*π*_*i* = 2,*k*_) because it is better suited as a measure of intervention effect in clinical trials[21]. In any case, RR and Odds Ratio (OR) are numerically easily interchangeable [22] using the expression 3. Additionally, the user can determine the component that drives the main effect of the therapy or, in other words, the RE. If no outcome is indicated, Bin-CE automatically assigns the label of RE to the outcome that requires the lowest SSR (Fig 2).

$${RR}_{k}=\frac{\pi_{2k}}{\pi_{1k}}=\frac{{OR}_{k}}{\left(1-\pi_{1k})+({OR}_{k}\pi_{1k}\right)};\;{OR}_{k}=\frac{{RR}_{k}(1-\pi_{1k})}{\left(1-{RR}_{k}\pi_{1k}\right)}$$ (3)

#### Step 1.2: Input data (Screen 2: Joint probability)

**Fig 3:**
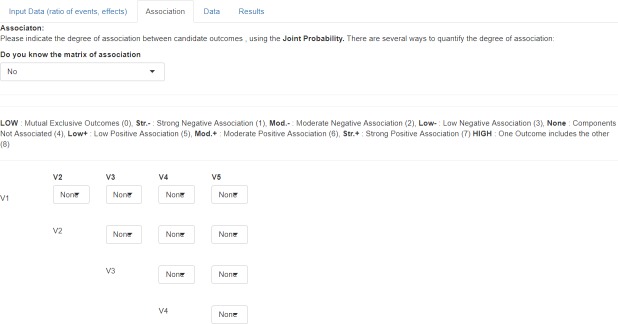
Data showed on screen 2: Joint probability (second-order association). On the second screen (Association) the known joint probabilities for the simultaneous occurrence of each pair of outcomes should be declared and their values uploaded in the appropriate cell. The semi-parametric approximations according to the Fréchet Bounds are employed for the unknown associations.

Bin-CE uses the joint probability as a parameter to quantify the degree of association between outcomes (i.e. second-order association). If values of joint probabilities between outcomes were known by the user they can be assigned manually. However, as usually their exact values are unknown, Bin-CE permits to enter semi-parametric approximations based on the Fréchet bounds (Fig 3). In this option, nine different scenarios are considered depending on the assumed magnitude and direction of the association between outcomes. One scenario considers that outcomes are non-associated. In this case Bin-CE internally assigns the association for independent events (i.e. *π*_*kk*′_ = *π*_*ik*_*π*_*ik*′_). Additionally, there are two specific scenarios, one for the maximum and another for the minimum possible association (i.e. minimum and maximum Fréchet bound). For instance, the association between the outcomes “CV death” and “Death from any cause” is the maximum possible association (i.e. all CV death can be considered as “death from any cause”) whereas the association between “ischemic stroke” and “hemorrhagic stroke” is the minimum possible association (i.e. they can be considered as mutually exclusive). There are also three scenarios indicating negative association: strong, moderate, and low negative association, and three scenarios indicating positive association (i.e. strong, moderate, and low positive association).

Bin-CE imputes the joint probability using: a) the marginal prevalence of each outcome (*π*_*ik*_,*π*_*ik*′_); b) the range between minimum and maximum of the Fréchet bound (*High*(*π*_*ik*_,*π*_*ik*′_)−*Low*(*π*_*ik*_,*π*_*ik*′_)); and finally, c) the non-association scenario (*π*_*kk*′_ = *π*_*ik*_*π*_*ik*′_). In (4) it is shown the mathematical expression applied when the user considers that outcomes are not associated, whereas in (5) and (6) the expressions for the negative and positive association are displayed.

$$\pi_{kk\prime }=\pi_{ik}\pi_{ik\prime }$$ (4)

$$\pi_{kk\prime }=\pi_{ik}\pi_{ik\prime }-\left(\frac{\left[\pi_{kk^{\prime }}-Low(\pi_{ik},\pi_{ik^{\prime }})\right]}{3}\right)\Theta$$ (5)

$$\pi_{kk\prime }=\pi_{ik}\pi_{ik\prime }+\left(\frac{\left[High(\pi_{ik},\pi_{ik^{\prime }})-\pi_{kk^{\prime }}\right]}{3}\right)\Theta$$ (6)

The parameter Θ takes value 1 for low, 2 for moderate, and 3 for strong positive/negative associations. The user can combine exact joint probabilities for some pairs of outcomes and semi-parametric approximations for other pairs.

**Fig 4:**
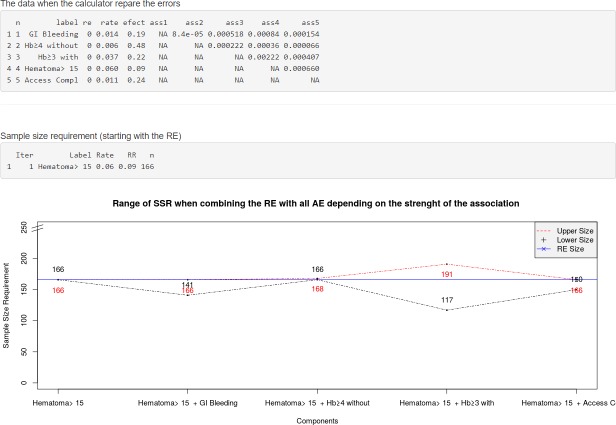
Data showed on Screen 3: Checking upload data and firsts results. On the third screen (Data) the user can check the data uploaded (i.e. labels, the RE, the rate of events in the control groups, the effect of the therapy and a triangular-matrix with all the pairs of joint probabilities). On this screen the SSR for the RE or for the outcome with a minimum SSR is shown. Finally, Bin-CE depicts a plot with the range of SSR when combining the RE with each of the other AE assuming the lower and the higher association of the Fréchet Bounds.

On the third screen (Fig 4), Bin-CE shows the input data. Graphically, Bin-CE shows on this screen a plot with the potential increase or decrease in the SSR when the RE is combined with each one of the other additional outcomes, but without taking into account the degree of association considered. The plot depicts the SSR for both the minimum and maximum association for each pair of outcomes with the RE. This plot can be useful to determine the impact of the strength of association on the SSR. Also, on this screen, Bin-CE shows the SSR for the RE.

#### Step 2: An iterative algorithm

In the first iteration of the algorithm, Bin-CE computes the SSR of the RE (SSR_RE_) and the (k-1) possible SSRs of the hypothetical two-components CEs when combining the RE and the other AE (SSR_k = 2 … K_). From all possible additional outcomes Bin-CE selects the one that combined with the RE leads to the largest decrease in the SSR_RE_. Then, this combination of the RE and the AE with the lowest SSR is considered as the new RE and the algorithm starts again seeking among the k-2 remaining outcomes. Bin-CE additionally estimates the rate of the CE (1) and the effect of the CE on the treatment group (the methodology to compute these parameters has been discussed previously [7,14,16]).

**Fig 5:**
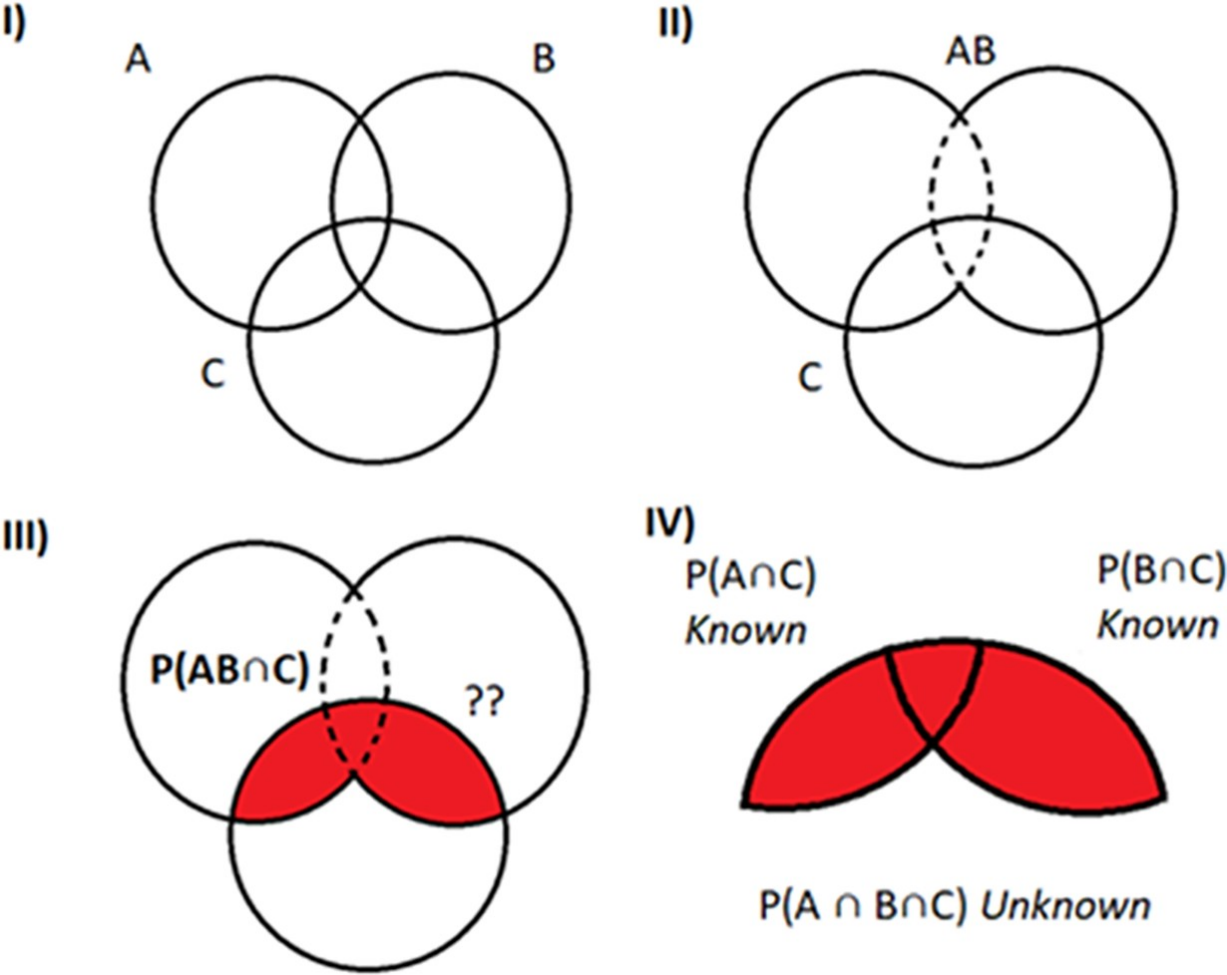
**Second and third-order associations between three hypothetical outcomes (A, B and C).** The association between a new hypothetical CE obtained by combination of outcomes A and B and the outcome C (red-colored probability) is the result of combining the joint probability between the pairs of outcomes A and C and the outcomes B and C. The value of this probability is (1): Prob((X_AC_ = 1)∩(X_BC_ = 1)) = π_AC_+π_BC_−π_ABC_≈π_AC+_π_BC_−π_AC_*π_BC_. Then Bin-CE estimates the unknown joint probability among the 3 outcomes π_ABC_ with the product of both probabilities (i.e. π_AC_*π_BC_). Although this is only an approximation, the potential error should be small since the real proportion of patients with the 3 outcomes has to be within the Fréchet Bounds (max{0;π_AC_+π_BC_−1}≤π_ABC_≤min{π_AB_,π_BC_}).

The joint probability between the new RE and all other AE candidates has now to be recalculated. Fig 5, shows three hypothetical outcomes (A, B and C). In this example the outcomes A and B combined in a CE (CE_AB_) can decrease the SSR of the initial RE (i.e. A outcome). Then the joint probability between the CE_AB_ and the outcome C (i.e. a second order association) is red-colored. The joint probability between AC and BC is known or have been estimated using the semi-parametric approach, but the joint probability between the three outcomes (i.e. the third-order association: *π*_*ABC*_) is unknown. To solve this issue, it is assumed a non-associated scenario as a good approximation of the third-order association, *π*_*ABC*_ = *π*_AC_*π_*AB*_.

If a new CE of three components with lower SSR that the CE of two components exists, this new three-components-CE is in turn considered as the new provisional RE, and the algorithm starts the process again until either all k-1 AE are included in the CE or there is no additional gain in the SSR.

#### Step 3: Results (Screen 4: Table and plots output)

**Fig 6:**
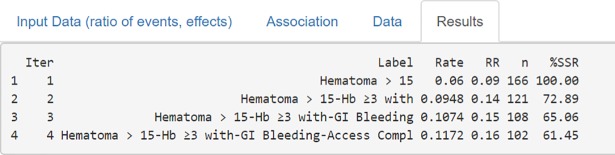
Data showed on screen 4: Results (table). Each line presents the CE selected in each step, specifically: the label of the combined components, the incidence rate and the Relative Risk, the SSR (number of subjects required in each treatment group) and the proportion of SSR compared to that used for the isolated RE.

**Fig 7:**
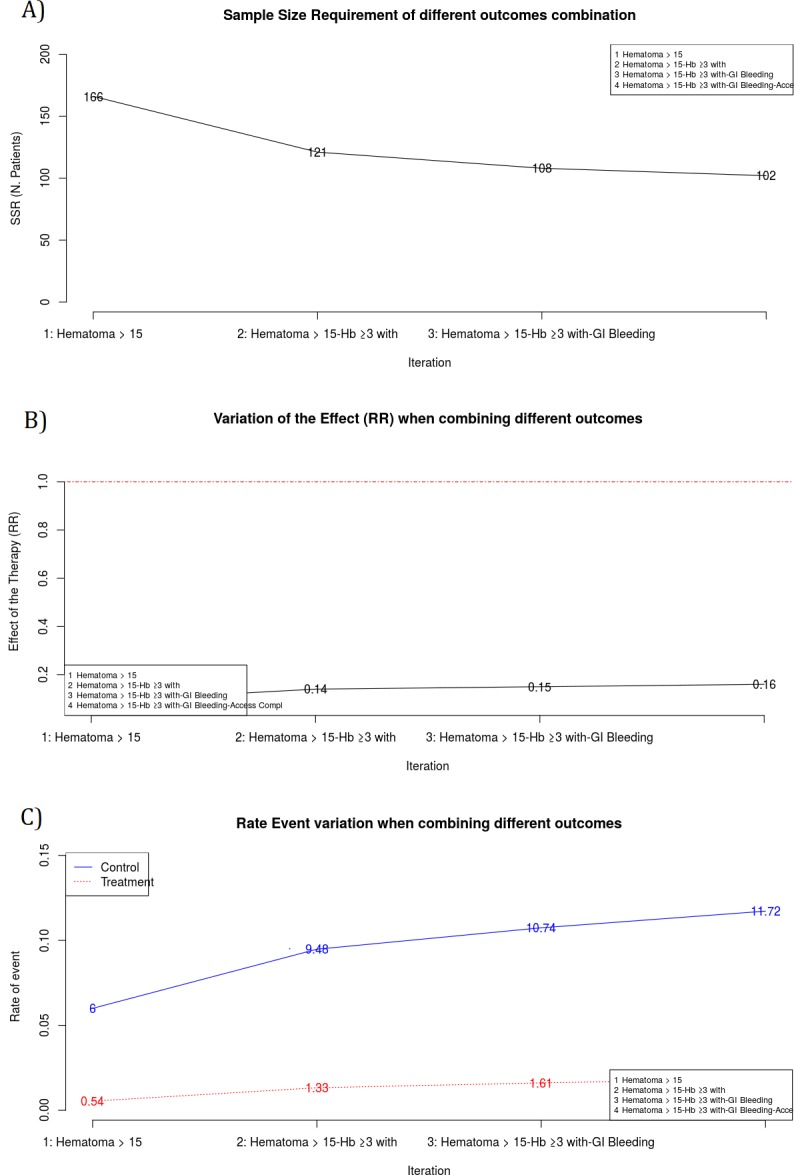
Data showed on screen 4. **Plots of the main results. A** Sample Size Requirement in each iteration. Y-axis is ranged from zero and it represents the SSR computed within each iteration. X-axis shows each one of the Bin-CE iterations. In the example, first iteration corresponds to the RE “Hematoma > 15 cm.”, with SSR of 166. The second iteration corresponds to the CE “Hematoma > 15 cm” and “Hb drop ≥3 g/dl with overt bleeding”, with a SSR of 121. Finally Bin-CE proposes to combine 4 outcomes to the last CE, in this case the SSR is of 102. B Effect of the therapy in each Bin-CE iteration. In the example, the effect of the therapy was a RR of 0.09 in the first iteration and it increased (decreased the efficacy) until 0.16 at the last iteration of Bin-CE. **C** Rate of events in each iteration. This plot presents, for each Bin-CE iteration, the rate of events for both the control group and the treatment group. In the example, the rate of event for the control and treatment group in the first iteration were 6% and 0.54% respectively, which increased to 9.48% and 1.33% in the second iteration. Finally, a small increase in rates was achieved through the third and fourth iteration, in agreement with the small reduction in SSR displayed in A.

Bin-CE shows a table that includes the best combination of outcomes, the rate of events, the effect of the therapy on the CE, the SSR estimated and the percentage of SSR reduction achieved in each step (Fig 6). It also presents several figures showing sequential changes of both SSR, event rates and intervention effect (RR) at each iteration (Fig 7).

### Numerical example

The STEMI-RADIAL study [23] is a randomized, multicenter, parallel group trial. Patients that were admitted with an acute STEMI, within 12 h of symptom onset, and referred for an invasive approach were randomized to a radial or a femoral approach. The underlying hypothesis was that radial approach is safer than femoral approach.

The primary endpoint was the cumulative incidence of major bleeding and vascular access site complications requiring intervention at 30 days. The components of the CE were: gastrointestinal bleeding, hemoglobin drop ≥ 4g/dl without overt bleeding, hemoglobin ≥ 3g/dl with overt bleeding, hematoma > 15 cm, transfusion (non-coronary artery bypass graft), and vascular access complication. Secondary outcomes at 30 days include Major Adverse Cardiovascular Event, defined as combinations of death, myocardial infarction, stroke and coronary artery bypass graft. Since STEMI-RADIAL study included both ischemic and bleeding outcomes we illustrate the performance of Bin-CE using separately both set of outcomes (Table 1). Figs 2, 3, 4, 6 and 7 refers to the bleeding set of outcomes.

**Table 1:** Primary and secondary outcomes in STEMI-RADIAL clinical trial.

	Radial (n = 348)		Femoral (n = 359)		Effect^b^		
	n	%	n	%	RR	OR	Diff
**Ischemic Outcomes**							
Death	8	2.30%	11	3.06%	1.33	1.34	0.77%
Infarction	4	1.15%	3	0.84%	0.73	0.72	-0.31%
Stroke	1	0.29%	1	0.28%	0.97	0.97	-0.01%
CABG/Revascularization	32	9.20%	28	7.80%	0.85	0.84	-1.40%
**Bleeding Outcomes**							
GI Bleeding	5	1.44%	1	0.28%	0.19	0.19	-1.16%
Hb drop ≥ 4 g/dl without overt bleeding	2	0.57%	1	0.28%	0.48	0.48	-0.30%
Hb drop ≥ 3gd/dl with overt bleeding	13	3.74%	3	0.84%	0.22	0.22	-2.90%
Hematoma > 15 cm.	21	6.03%	2	0.56%	0.09	0.09	-5.48%
Transfusion^a^	3	0.86%	0	0.00%	0.00	0.00	-0.86%
Vascular Access Complication	4	1.15%	1	0.28%	0.24	0.24	-0.87%

^a^: Transfusion was excluded for the analysis because none case in Femoral group was observed. In this particular case, the effect is unlikely.

^b^: The effect is presented as the ratio between the incidences (risk ratio), using Odds Ratio see expression (3) and by the difference of incidences. Bin-CE uses the Risk Ratio

GI: Gastrointestinal, Hb: Hemoglobin, Diff: Difference of incidences, RR: Risk Ratio, OR: Odds Ratio.

Using data from STEMI-RADIAL trial, we illustrate the selection of the most efficient combination among the five bleeding outcomes (i.e. 31 possible combinations) and the 4 ischemic outcomes (i.e. 15 possible combinations) in two CEs, one for the bleeding outcomes and the ischemic outcomes, employing Bin-CE.

## Results

The association between each pair of components is not available in the STEMI-RADIAL publication. Although it is highly recommended, the report of the association between individual components is generally inadequate [2,24,25]. Since the present paper has an educational purpose we assume absence of association among individual outcomes of the STEMI-RADIAL clinical trial. Bin-CE imputes the product of both probabilities when non-associations scenario is clicked (i.e. see Eq (4)).

### Bin-CE workflow

In a first step (Fig 2), the user introduces the assumed rate of events in the radial group, the assumed effect of the therapy, and the degree of association between each pair of outcomes (Fig 3). If the exact degree of association between two pairs of outcomes is unknown, the semi-parametric approach is employed. Then, Bin-CE returns the joint probabilities between each pair of component outcomes (Fig 4). In the bleeding example, associations between components are unknown. In this example, for simplicity it is assumed that outcomes are not associated.

Bin-CE plots the potential SSR using the different combinations between the RE and each AE (see bottom of the Fig 4). Note that the exact value of the association is not implemented in this plot. Rather the plot shows the potential SSR variation for each combination of outcomes considering the whole range of joint probabilities according to the Fréchet bounds. In the example, the SSR of the CE “Hematoma > 15 cm or Hb drop ≥3gd/dl with overt bleeding” could be as low as 117 or as high as 191 in case of minimum and maximum degree of association respectively. Any component can be declared as RE; in the example ‘Hematoma > 15 cm’ does this role. By defect Bin-CE considers the component which needs a minimum SSR as the RE.

Figs 6 and 7A show the CE with the lowest SSR. In this case the combination of ‘Hematoma > 15 cm’, ‘hemoglobin ≥ 3g/dl with overt bleeding, ‘gastrointestinal bleeding and ‘vascular access complication’ would have provided the lowest SSR (n = 102 per group). Moreover, using this combination, a 38.55% (i.e. 61.45% of the initial SSR) reduction of the SSR employing the primary outcome “Hematoma > 15 cm.” would have been achieved (166 vs. 102). It is also shown that the combination of “Hematoma > 15 cm” and “hemoglobin ≥ 3g/dl with overt bleeding” contributes the most to SSR, achieving a reduction of 27.11%. Therefore, although the addition of the outcome “gastrointestinal bleeding” or “vascular access complication” further reduces the SSR, the magnitude of this reduction is so small (i.e. from 121 to 108 and from 108 to 102) that its inclusion in the CE could be debatable. The other outcomes are not selected by Bin-CE because when their inclusion does not reduce the SSR. Fig 7B and 7C show the variation on the rate of events and the effect of therapy, respectively, at each iteration.

#### Sensitivity analysis

Since the actual strength of association between some outcomes is unknown and cannot be easily inferred, an additional analysis has been performed considering combinations of the all nine possible semi-parametric values of the strength of association between the five outcomes of the trial (Tables 2 and 3).

**Table 2:** Sensitivity analysis. Bin-CE results when the strength of associations varies from the lowest to the highest possible values. Bleeding Outcomes.

*Strength of* *Association*	*CE*	*#Components*	*Rate of* *Events*	*Risk* *Ratio*	*SSR*	*%*
Lowest	*Hematoma> 15-Hb≥3 with-GI Bleeding-Access Compl-Hb≥4*	5	12.80%	0.18	97	58.43%
Strong Negative	*Hematoma> 15-Hb≥3 with-GI Bleeding*	3	11.01%	0.15	105	63.25%
Moderate Negative	*Hematoma> 15-Hb≥3 with-GI Bleeding*	3	10.92%	0.15	106	63.86%
Low Negative	*Hematoma> 15-Hb≥3 with-GI Bleeding-Access Compl*	4	11.84%	0.16	101	60.84%
No Association	*Hematoma> 15-Hb≥3 with-GI Bleeding-Access Compl*	4	11.72%	0.16	102	61.45%
Low Positive	*Hematoma> 15-Hb≥3 with-GI Bleeding-Access Compl*	4	9.40%	0.14	122	73.49%
Moderate Positive	*Hematoma> 15-Hb≥3 with-GI Bleeding*	2	7.74%	0.14	149	89.76%
Strong Positive	*Hematoma> 15-GI Bleeding*	2	6.33%	0.10	160	96.39%
Highest	*Hematoma> 15*	1	6.00%	0.09	166	100%

Hb: Hemoglobin, GI: Gastrointestinal.

**Table 3:** Sensitivity analysis. Bin-CE results when the strength of associations varies from the lowest to the highest possible values. Ischemic Outcomes.

*Strength of Association*	*CE*	*#Components*	*Rate of* *Events*	*Risk* *Ratio*	*SSR*	*%*
Lowest	*CABG-Infarction*	2	10.35%	0.84	4724	73.62%
Strong Negative	*CABG-Infarction*	2	10.32%	0.84	4771	74.35%
Moderate Negative	*CABG-Infarction*	2	10.30%	0.84	4819	75.10%
Low Negative	*CABG-Infarction*	2	10.27%	0.84	4867	75.85%
No Association	*CABG-Infarction*	2	10.24%	0.84	4917	76.62%
Low Positive	*CABG-Infarction*	2	9.98%	0.84	5230	81.50%
Moderate Positive	*CABG-Infarction*	2	9.72%	0.84	5580	86.96%
Strong Positive	*CABG-Infarction*	2	9.46%	0.85	5973	93.08%
Highest	*CABG*	1	9.20%	0.85	6417	100%

CABG: coronary artery bypass graft.

Table 2 presents the sensitivity analysis for bleeding outcomes and Table 3 the sensitivity analysis for the ischemic outcomes. For the bleeding outcomes, the strength of association is more determinant than in the ischemic example in terms of SSR. So the strength of association determines the set of events to combine. Thus, for the bleeding events, as the magnitude of positive association decreases the number of combined outcomes rapidly increases, being the lowest magnitude of association (i.e. disjoint outcomes) the most favorable scenario for the CE (SSR = 97). In summary, for the bleeding events the strength of associations between outcomes has a strong influence on both the number of bleeding outcomes to be combined in the CE and SSR reductions (from 166 to 97, representing the 58% of the initial SSR).

On other hand, the strength of association hardly determines the number of components combined in the ischemic CE. In this case the outcomes ‘coronary artery bypass graft’ and ‘myocardial infarction’ are consistently gathered in a CE regardless the degree of association, except for the scenario with the highest degree of association where Bin-CE proposes only use a single outcome. As Table 3 shows the influence of the degree of association on the SSR is modest for the ischemic events (i.e. a maximum reduction of 26% on ischemic example vs. a 42% reduction in the bleeding example).

The sensitivity analysis not only has to be considered for the degree of association between events. For example, in STEMI-RADIAL Trial the effect of some events could not be estimated accurately (i.e. Hb. drop ≥ 4 g/dl without overt bleeding). A small number of patients had the event, and in this situation the variability of the effect can be high. Thus, simulating different scenarios with different effects as a sensitivity analyses is advisable because it will provide robustness.

## Discussion

As long as the use of CEs in clinical trials increases steadily, the need for simple and robust methods for a comprehensive use of this tool arises. We present a computational method to guide decisions concerning the optimal choice of the number of dichotomous outcomes to be combined in a binary CE in order to minimize the SSR. It also permits to explore a variety of plausible scenarios by varying the assumed strength of association between the outcomes, which can be useful to evaluate the potential usefulness of using CE in each clinical situation. Eventually this could avoid the futile recruitment of patients in clinical trials, which will benefit the researches, patients, and the scientific community. However, the time-to-event analysis can be approximated though working with the probability of event by a certain follow-up time as a binary outcome, so the sample should not be very different with both approximations. Although there are many publications addressing the statistical methods to analyze the data from clinical trials using CEs, the problem of the sample size computation and its relationship with the strength of association between components of a binary CE has not been addressed in depth [7,12,15,17,26,27]. Sozu *et al*.[7] reported the mathematical approach to estimate the SSR of a CE and our group[16] explored how the strength of association between the 2 components of a CE can affect this SSR. In the field of survival analysis, Gómez *et al*. have reported several findings concerning the SSR when using CE in the time-to-event setting [15,28–31]. In this manuscript we present *Bin-CE*, a free intuitive Shiny App available at https://uesca-apps.shinyapps.io/bincep/ with the objective of computing the SSR of a binary CE with more than two potentially candidate endpoints. It has to be noted that the iterative algorithm developed to compute the SSR is not an exact method. Thus, some degree of error is assumed because of the categorical approximation of the second-order associations and the imputation of the third-order association using the mathematical expectation. However, the exact solution would imply the estimation of 2^*k*^−1 coefficients (for instance, for PARADIGHM-HF study [32], with 5 outcomes it should be necessary to estimate 31 parameters), which is not practical in most situations. However, our sensitive analyses assuming different strengths of association between outcomes indicate that the potential SSR error is not relevant from the clinical point of view (Table 2).

Bin-CE has been designed to help the applied trialist so we have tried the software to be intuitive and easily manageable. In a first step, the user determines the number of component candidates and the prespecified type I and II error rates. Secondly, the user assigns the labels and inputs the prevalence and the assumed effect of the therapy. Finally, the users have to include the degree of the association between components, which is obviously a hard issue because in most cases this parameter is unknown. In this sense, the App allows to introduce the exact association (i.e. the joint probability) if it is known, which in most cases will not be the case, or to use a semi-parametric approximation based on the Fréchet bounds otherwise. The semi-parametric approximation is, in our view, a pragmatic solution, since for clinicians it is usually easy to intuitively know whether components are correlated or not and, in the case of correlation, if it is positive or negative and the rough strength of the association.

As a limitation, the reliability of Bin-CE lies on the validity of the assumption that the mathematical expectation (product of probabilities) is a good approximation of the third-order associations between outcomes. However, as it is shown in the sensitivity analysis, the potential error considering different levels of associations is unlikely to be clinically relevant.

Bin-CE uses a greedy step-wise forward iterative algorithm to find the optimal combination. So, Bin-CE solves locally the problem (i.e. to combine the RE with the AE whom establish the lowest SSR) in each step. However, although we assume that the final combination of outcomes in a CE selected by Bin-CE is the most efficient, it cannot be demonstrated. Such demonstration would require simulating all possible combinations from all available outcomes, which would overcharge the programme.

In this article, we have focused on the issue of improving efficiency in clinical trials through the minimization of SSR, provided there are a set of potential candidate outcomes available. However, the importance of other critical considerations in the decision to use a CE (clinical relevance of single components, homogeneity of intervention effects, interpretability of results, etc.) should not be underemphasized[3–5].

There are several types of CE depending on the type of outcomes to be combined. Time-to-event CE are by far the most used CE in clinical trials, although there are many other possibilities (i.e. multivariate normal outcomes or multinomial variables as Likert scales). In this sense, binary outcomes are used when the outcomes occur in a short-fixed follow-up period or when the outcome has to be dichotomized. Although binary CE are much less used in clinical trials, they are very useful to implement new knowledge on this topic because their mathematic properties are well-stablished. Other authors [7,28,33–35] have used binary CE to illustrate their proposes. In any case, a clear limitation of the present paper is that our findings cannot be extrapolated directly to time-to event analyses.

In spite of that, we believe that our work is conceptually valid also in survival analysis in the sense that an influence of the outcomes association and of the prevalence on the sample size requirement is also required [15,29,36].

Bin-CE selects the best set of outcomes comparing the SSR for the RE versus the combination of RE with one of the AE remaining. The SSR is computed considering the simplest hypothesis testing case of equality of probabilities. This approximation is simpler enough when the number of outcomes to be combined is either large (i.e. multiplicity testing problem) or other type of hypotheses testing is desired (e.g. at least one of the components is significant). Some authors have studied in depth[33,37–39], the problem of multiple testing and the heterogeneity of possible contrasts applied giving some recommendations to handle it. We share the source code (Supplementary Material 2) in order to adapt Bin-CE code to other particular specifications.

An essential difference between Bin-CE and other tools to compute SSR is that Bin-CE calculates the SSR for the most efficient CE and not for other prespecified CEs. Thus, the clinical trialists have to be aware that bin-CE does not take into account the clinical relevance of the outcomes combinates, which must always be assessed by the researcher. In this paper, we describe a new tool that could be useful to reduce the SSR when one is considering a CE as a primary endpoint. However, not only the SSR but other issues must also be taken into consideration when using a CE in order to correctly interpret the final CE. We and other authors have address these issues previously [3,5,6,25,39–44].

So, although Bin-CE does not solve completely the SSR in CE issue, it can be considerate a first step. In this sense, we encourage other researchers to improve the utility of this tool.

To summarize, in this manuscript a free tool to estimate the SSR in a two-arms randomized clinical trial using a binary CE with more than two-components is presented. When a set of possible candidate outcomes is to be combined in a CE and the decision to combine them or not depends on the SSR Bin-CE can be a good tool to consider. Accessible at: https://uesca-apps.shinyapps.io/bincep/.

## Supporting information

S1 File List of coefficients used to assess the association between two binary variables. (DOCX)

S2 File Source code of Bin-CE. This file includes all functions used in Bin-CE but it not includes the appearance of the tool.
(R) Click here for additional data file.

S3 File How Bin-CE runs: The mathematical functions. (DOCX)
