# Supplementary material for: Bin-CE: A comprehensive web application to decide upon the best set of outcomes to be combined in a binary composite endpoint
Source: PLoS One. 2018 Dec 13;13(12):e0209000. doi: 10.1371/journal.pone.0209000 (PMC6292611; doi:10.1371/journal.pone.0209000)
Supplement: S1 File — (DOCX) [file pone.0209000.s001.docx]

**Supplementary Material – List of Coefficients used to assess the association between two binary variables.**

When a composite endpoint (CE) is defined using two binary components $(X_{1}=Relevant Endpoint, X_{2}=Additive Endpoint)$, a 2x2 contingency table summarizes all the information.

|  | ***Additive Endpoint -*** $\boldsymbol{X}_{\boldsymbol{2}}$ | | |
| --- | --- | --- | --- |
| ***Relevant Endpoint -*** $\boldsymbol{X}_{\boldsymbol{1}}$ | *Yes {1}* | *No {0}* | Total |
| *Yes {1}* | $a$ | b | $a+b=p_{1}$ |
| *No {0}* | $c$ | d | ${c+d=q}_{1}$ |
| Total | ${a+c=p}_{2}$ | $b+d=q_{2}$ | $n$ |

***a*** *= number or proportion of patients for which both events (RE and AE) occur.* ***b*** *= number or proportion of patients for which occurs RE but not AE.*

***c*** *= number or proportion of patients for which occurs AE but not RE.****d*** *= number or proportion of patients for which occurs neither RE nor AE.*

$\boldsymbol{p}_{\boldsymbol{1}}\boldsymbol{=a+b}$ *= number or proportion of patients for which occur RE.* $\boldsymbol{q}_{\boldsymbol{1}}\boldsymbol{=c+d}$ *= number of proportion of patients for which not occur RE.*

$\boldsymbol{p}_{\boldsymbol{2}}\boldsymbol{=a+c}$ *= number or proportion of patients for which occur AE.* $\boldsymbol{q}_{\boldsymbol{2}}\boldsymbol{=b+d}$ *= number of proportion of patients for which not occur AE.*

With the following 3 parameters ($\pi_{1}{,\pi_{2},\pi}_{12}$) the bivariate distribution is fully defined(1). The reader must note that the value $p_{1}$ in the last table is interchangeable for $\pi_{1}$, $p_{2}$ for $\pi_{2}$ and p_12_ for $\pi_{12}$. So, knowing the prevalence of RE, AE and the prevalence of patients in which occur both events, the table can be rewrite as:

|  | ***Additive Endpoint -*** $\boldsymbol{X}_{\boldsymbol{2}}$ | | |
| --- | --- | --- | --- |
| ***Relevant Endpoint -*** $\boldsymbol{X}_{\boldsymbol{1}}$ | *Yes {1}* | *No {0}* | Total |
| *Yes {1}* | $\pi_{12}$ | $\pi_{1}-\pi_{12}$ | $\pi_{1}$ |
| *No {0}* | $\pi_{2}-\pi_{12}$ | $1-\pi^{*}$ | ${1-\pi}_{1}$ |
| Total | $\pi_{2}$ | ${1-\pi}_{2}$ | $1$ |

$\pi_{1}$*= marginal prevalence for the RE,* $\pi_{2}$*= marginal prevalence for the AE.* $\pi_{12}$*= prevalence of the intersection.* $\pi_{i}^{*}$ *= prevalence of CE* $\pi^{*}=P\left( X^{*} \right)=\pi_{1}{+\pi}_{2}-\pi_{12}$

The corresponding 2x2 contingency table for the study PARADIGM-HF(2) which put the association between two outcomes (i.e. Fatal Myocardial Infarction and Non-Fatal Myocardial Infarction), would be:

| **PARADIGM-HF study** | ***Additive Endpoint – Non Fatal MI*** | | |
| --- | --- | --- | --- |
| ***Relevant Endpoint – Fatal MI*** | *Yes {1}* | *No {0}* | Total |
| *Yes {1}* | $415 [4.9\%]$ | 836 $[10.0\%]$ | $1,251$ [14.9%] |
| *No {0}* | $780 [9.3\%]$ | $6368 [75.8\%]$ | $7148 [85.1\%]$ |
| Total | $1,195 [14.2\%]$ | $7204 \left[ 85.8\% \right]$ | $8,399 \left[ 100\% \right]$ |

This appendix presents an extract of the work done by Warrens M.J. (3–6), in his thesis “Similarity Coefficients for Binary Data” . It is ordered chronologically on the year of appearance. We have summarized the author, the formulae, the bibliographic reference, the assessment on PARADIGM-HF study example and whether these coefficients were used in our paper and the reason why we have chosen them. Although this list is very extensive it is not exhaustive. Other indicators published on the literature can be found in (7).

| Id | Indicator | Author/Year | Similarity Coefficient | Value in  PARADIGM-HF Study |
| --- | --- | --- | --- | --- |
| 1 | Peir1, Peri2 | Peirce (1884) (8) | $S_{Peir1}=\frac{ad-bc}{p_{1}\left( 1-p_{1} \right)}=\frac{\pi_{12}-\pi_{1}\pi_{2}}{\pi_{1}\left( 1-\pi_{1} \right)}$  $S_{Peir2}=\frac{ad-bc}{p_{2}(1-p_{2})}=\frac{\pi_{12}-\pi_{1}\pi_{2}}{\pi_{2}\left( 1-\pi_{2} \right)}$ | $S_{Peir1}=0.223$  $S_{Peir2}=0.231$ |
| 2 | Doo | Doolittle (1885) (9), Pearson (1926) (10) | $S_{Doo}=\frac{{(ad-bc)}^{2}}{p_{1}q_{1}p_{2}q_{2}}=\frac{\left( \pi_{12}-\pi_{1}\pi_{2} \right)^{2}}{\pi_{1}\left( 1-\pi_{1} \right)\pi_{2}\left( 1-\pi_{2} \right)}$ | $S_{Doo}=0.051$ |
| 3 | Yule1 | Yule (1900)(11), Montgomery and Crittenden (1977)(12) | $S_{Yule1}=\frac{ad-bc}{ad+bc}=\frac{\pi_{12}-\pi_{1}\pi_{2}}{\pi_{12}+\pi_{1}\pi_{2}+2\pi_{12}(\pi_{12}-\pi_{1}-\pi_{2})}$ | $S_{Yule1}=0.604$ |
| 4 | Chi-square | Pearson (1905), quoted by Yule and Kendall (1950) (13) | $x^{2}=\frac{n{(ad-bc)}^{2}}{p_{1}q_{1}p_{2}q_{2}}=\frac{n\left( \pi_{12}-\pi_{1}\pi_{2} \right)^{2}}{\pi_{1}\left( 1-\pi_{1} \right)\pi_{2}\left( 1-\pi_{2} \right)}$ | $x^{2}=432.3$ |
| 5 | Forbes | Forbes (1907) (14) | $S_{Forbes}=\frac{na}{p_{1}p_{2}}=\frac{\pi_{12}}{\pi_{1}\pi_{2}}$ | $S_{Forbes}=2.332$ |
| 6 | JI | Jaccard (1912) (15) | $S_{JI}=\frac{a}{a+b+c}=\frac{\pi_{12}}{\pi^{*}}$ | $S_{JI}=0.204$ |
| 7 | Phi | Yule (1912) (16), Pearson & Heron (1913) (17) | $S_{\rho}=\frac{ad-bc}{\sqrt{p_{1}q_{1}p_{2}q_{2}}}=\frac{\pi_{12}-\pi_{1}\pi_{2}}{\sqrt{\pi_{1}\left( 1-\pi_{1} \right)\pi_{2}\left( 1-\pi_{2} \right)}}$ | $S_{\rho}=0.227$ |
| 8 | Yule2 | Yule (1912) (16) | $S_{Yule2}=\frac{\sqrt{ad}-\sqrt{bc}}{\sqrt{ad}+\sqrt{bc}}=\frac{\sqrt{\pi_{12}(1-\pi^{*})}-\sqrt{({\pi_{1}-\pi}_{12})({\pi_{2}-\pi}_{12})}}{\sqrt{\pi_{12}(1-\pi^{*})}+\sqrt{({\pi_{1}-\pi}_{12})({\pi_{2}-\pi}_{12})}}$ | $S_{Yule2}=0.336$ |
| 9 | SD | Gleason (1920) (18), Dice (1945) (19),  Sørenson (1948) (20,21), Nei and Li (1979) (22) | $S_{SD}=\frac{2a}{p_{1}{+p}_{2}}=\frac{2\pi_{12}}{\pi_{1}{+\pi}_{2}}$ | $S_{Gleas}=0.339$ |
| 10 | Mich | Michael (1920) (23) | $S_{Mich}=\frac{4(ad-bc)}{{(a+d)}^{2}+{(b+c)}^{2}}=\frac{4(\pi_{12}-\pi_{1}\pi_{2})}{{(\pi_{12}+(1-\pi^{*}))}^{2}+{(({\pi_{1}-\pi}_{12})({\pi_{2}-\pi}_{12}))}^{2}}$ | $S_{Mich}=0.164$ |
| 11 | KU1, KU2 | Kulcynski (1927) (24), Driver & Kroeber (1932) (25) | $S_{KU1}=\frac{1}{2}\left( \frac{a}{p_{1}}+\frac{a}{p_{2}} \right)=\frac{1}{2}\left( \frac{\pi_{12}}{\pi_{1}}+\frac{\pi_{12}}{\pi_{2}} \right)$  $S_{KU2}=\frac{a}{b+c}=\frac{\pi_{12}}{\pi^{*}-\pi_{12}}$ | $S_{KU1}=0.340$  $S_{KU2}=0.257$ |
| 12 | BB | Braun-Blanquet (1932) (26) | $S_{BB}=\frac{a}{max\left( p_{1}{,p}_{2} \right)}=\frac{\pi_{12}}{max\left( \pi_{1}{,\pi}_{2} \right)}$ | $S_{BB}=0.332$ |
| 13 | OC | Driver & Kroeber (1932) (25), Ochiai (1957) (27), Fowlkes and Mallows (1983) (28) | $S_{OC}=\frac{a}{\sqrt{p_{1}p_{2}}}=\frac{\pi_{1}}{\sqrt{\pi_{1}\pi_{2}}}$ | $S_{OC}=0.339$ |
| 14 | KR | Kuder & Richardson (1937) (29), Cronbach (1951) (30) | $S_{KR}=\frac{4(ad-bc)}{p_{1}\left( 1-p_{1} \right){+p}_{2}\left( 1-p_{2} \right)+2(ad-bc)}=\frac{4(\pi_{12}-\pi_{1}\pi_{2})}{\pi_{1}\left( 1-\pi_{1} \right)+\pi_{2}\left( 1-\pi_{2} \right)+2(\pi_{12}-\pi_{1}\pi_{2})}$ | $S_{KR}=0.370$ |
| 15 | JP | Russel & Rao (1940) (31) – Intersection Probability | $S_{JP}={\frac{a}{a+b+c+d}=\pi}_{12}$ | $S_{RR}=0.049$ |
| 16 | JI* | Simpson (1943) (32,33) – Relative Jaccard Index or Overlap Coefficient | $S_{{JI}^{*}}=\frac{a}{min\left( p_{1},p_{2} \right)}=\frac{\pi_{12}}{min\left( \pi_{1},\pi_{2} \right)}$ | $S_{{JI}^{*}}=0.347$ |
| 17 | Dice1, Dice2 | Dice (1945) (19), Wallace (1983) (34), Post & Snijders (1993) (35) | $S_{Dice1}=\frac{a}{p_{1}}=\frac{\pi_{12}}{\pi_{1}}$  $S_{Dice2}=\frac{a}{p_{2}}=\frac{\pi_{12}}{p_{2}}$ | $S_{Dice1}=0.347$  $S_{Dice2}=0.332$ |
| 18 | Loe | Loevinger (1947,1948) (36,37), Mokken (1971) (38), Sijtsma & Molenaar (2002) (39) | $S_{Loe}=\frac{\left( ad-bc \right)}{min\left( p_{1}q_{2},p_{2}q_{1} \right)}=\frac{\left( \pi_{12}-\pi_{1}\pi_{2} \right)}{min\left( \pi_{1}\left( 1-\pi_{2} \right){;\pi}_{2}\left( 1-\pi_{1} \right) \right)}$ | $S_{Loe}=0.233$ |
| 19 | Cole1, Cole2 | Cole (1949) (40) | $S_{Cole1}=\frac{\left( ad-bc \right)}{p_{1}q_{2}}=\frac{\left( \pi_{12}-\pi_{1}\pi_{2} \right)}{\pi_{1}\left( 1-\pi_{2} \right)}$  $S_{Cole2}=\frac{\left( ad-bc \right)}{p_{2}q_{1}}=\frac{\left( \pi_{12}-\pi_{1}\pi_{2} \right)}{\pi_{2}\left( 1-\pi_{1} \right)}$ | $S_{Cole1}=0.231$  $S_{Cole2}=1.332$ |
| 20 | GK | Goodman & Kruskal (1954) (41) | $S_{GK}=\frac{2min\left( a,d \right)-b-c}{2\min\left( a,d \right)+b+c}=\frac{2min\left( \pi_{12},1-\pi^{*} \right)-\pi_{1}+\pi_{2}}{2\min\left( \pi_{12},1-\pi^{*},d \right)+\pi_{1}+\pi_{2}+2\pi_{12}}$ | $S_{GK}=0.999$ |
| 21 | Scott | Scott (1955) (42) | $S_{Scott}=\frac{4ad-\left( b+c \right)^{2}}{(p_{1}{+p}_{2})(q_{1}{+q}_{2})}=\frac{4\pi_{12}-\left( \pi_{1}+\pi_{2} \right)^{2}}{(\pi_{1}+\pi_{2})({(1-\pi}_{1})+(1-\pi_{2}))}$ | $S_{Scott}=0.227$ |
| 22 | SP | Sokal & Michener (1958) (43), Rand (1971) (44), Brennan & Light (1974) (45) – Simple Pair’s | $S_{SP}=\frac{a+d}{a+b+c+d}=\pi_{12}+1-\pi^{*}$ | $S_{SP}=0.808$ |
| 23 | Sorg | Sorgenfrei (1958) (46), Cheethan & Hazel (1969) (47) | $S_{Sorg}=\frac{a^{2}}{p_{1}p_{2}}=\frac{{\pi_{12}}^{2}}{\pi_{1}\pi_{2}}$ | $S_{Sorg}=0.115$ |
| 24 | Cohen | Cohen (1960) (48) | $S_{Cohen}=\frac{2(ad-bc)}{p_{1}q_{2}+p_{2}q_{1}}=\frac{2\left( \pi_{12}-\pi_{1}\pi_{2} \right)}{\pi_{1}\left( 1-\pi_{2} \right){+\pi}_{2}\left( 1-\pi_{1} \right)}$ | $S_{Cohen}=0.227$ |
| 25 | RT | Rogers & Tanimoto (1960)(49), Farkas (1978)(50) | $S_{RT}=\frac{a+d}{a+2\left( b+c \right)+d}=\frac{1-\pi_{1}-\pi_{2}+2\pi_{12}}{1+\pi_{1}+\pi_{2}-2\pi_{12}}$ | $S_{RT}=0.677$ |
| 26 | Sti | Stiles (1961) (51) | $S_{Sti}={log}_{10}\frac{n\left( \left\vert ad-bc \right\vert-\frac{n}{2} \right)^{2}}{p_{1}q_{1}p_{2}q_{2}}={log}_{10}\frac{\left( \left\vert\left( \pi_{12}-\pi_{1}\pi_{2} \right) \right\vert-\frac{1}{2} \right)^{2}}{\pi_{1}({1-\pi}_{1})\pi_{2}(1-\pi_{2})}$ | $S_{Sti}=2.634$ |
| 27 | Ham | Hamann (1961) (52), Holley & Guilford (1964) (53), Hubert (1977) (54) | $S_{Ham}=\frac{a-b-c+d}{a+b+c+d}=1-2\left( \pi_{1}+\pi_{2}-2\pi_{12} \right)$ | $S_{Ham}=0.615$ |
| 28 | Mount | Mountford (1962) (55) | $S_{Mount}=\frac{2a}{a\left( b+c \right)+2bc}=\frac{2\pi_{12}}{\pi_{12}\left( \pi_{1}+\pi_{2}-2\pi_{12} \right)+2\pi_{1}+{2\pi}_{2}-4\pi_{12}}$ | $S_{Mount}=0.000$ |
| 29 | FM | Fager & McGowan (1963) (56) | $S_{FM}=\frac{a}{\sqrt{p_{1}p_{2}}}-\frac{1}{2\sqrt{max\left( p_{1}{,p}_{2} \right)}}=\frac{\pi_{12}}{\sqrt{\pi_{1}\pi_{2}}}-\frac{1}{2\sqrt{max\left( \pi_{1}{,\pi}_{2} \right)}}$ | $S_{FM}=0.325$ |
| 30 | SS1, SS2, SS3, SS4 | Sokal and Sneath 1963 (57) | $S_{SS1}=\frac{a}{a+2\left( b+c \right)}=\frac{\pi_{12}}{\pi_{12}+2\left( \pi_{1}+\pi_{2}-2\pi_{12} \right)}$  $S_{SS2}=\frac{2 \left( a+d \right)}{2a+b+c+2d}=\frac{2-{2\pi}_{1}-{2\pi}_{2}+4\pi_{12}}{2-\pi_{1}-\pi_{2}+2\pi_{12}}$  $S_{SS3}=\frac{1}{4}\left( \frac{a}{p_{1}}+\frac{a}{p_{2}}+\frac{d}{q_{1}}+\frac{d}{q_{2}} \right)=\frac{1}{4}\left( \frac{\pi_{12}}{\pi_{1}}+\frac{\pi_{12}}{\pi_{2}}+\frac{(1-\pi^{*})}{(1-\pi_{1})}+\frac{(1-\pi^{*})}{(1-\pi_{2})} \right)$  $S_{SS4}=\frac{ad}{\sqrt{p_{1}p_{2}q_{1}q_{2}}}=\frac{\pi_{12}(1-\pi^{*})}{\sqrt{\pi_{1}\left( 1-\pi_{1} \right)\pi_{2}\left( 1-\pi_{2} \right)}}$ | $S_{SS1}=0.114$  $S_{SS2}=0.894$  $S_{SS3}=0.613$  $S_{SS4}=0.301$ |
| 31 | McC | McConnaughey (1964) (58) | $S_{McC}=\frac{a^{2}-bc}{p_{1}p_{2}}=\frac{\pi_{1}\pi_{12}+\pi_{2}\pi_{12}-\pi_{1}\pi_{2}}{\pi_{1}\pi_{2}}$ | $S_{McC}=-0.321$ |
| 32 | RG | Rogot & Goldberg (1966) (59) | $S_{RG}=\frac{a}{p_{1}+p_{2}}+\frac{d}{q_{1}+q_{2}}=\frac{\pi_{12}}{\pi_{1}+\pi_{2}}+\frac{1-\pi^{*}}{\left( 1-\pi_{1} \right)+(1-\pi_{2})}$ | $S_{RG}=0.613$ |
| 33 | John | Johnson (1967) (60) | $S_{John}=\frac{a}{p_{1}}+\frac{a}{p_{2}}=\frac{\pi_{12}}{\pi_{1}}+\frac{\pi_{12}}{\pi_{2}}$ | $S_{John}=0.679$ |
| 34 | HD | Hawkins & Dotson (1968) (61) | $S_{HD}=\frac{1}{2}\left( \frac{a}{a+b+c}+\frac{d}{b+c+d} \right)=\frac{1}{2}\left( \frac{\pi_{12}}{\pi^{*}}+\frac{1-\pi^{*}}{1-\pi_{12}} \right)$ | $S_{HD}=0.501$ |
| 35 | Fleiss | Fleiss (1975) (62) | $S_{Fleiss}=\frac{\left( ad-bc \right)\left( p_{1}q_{2}+p_{2}q_{1} \right)}{p_{1}{p_{2}q_{1}q}_{2}}=\frac{\left( \pi_{12}-\pi_{1}\pi_{2} \right)\left( \pi_{1}\left( 1-\pi_{1} \right)+\pi_{2}\left( 1-\pi_{2} \right) \right)}{\pi_{1}\left( 1-\pi_{1} \right)\pi_{2}\left( 1-\pi_{2} \right)}$ | $S_{Fleiss}=0.227$ |
| 36 | Clem | Clement (1976) (63) | $S_{Cle}=\frac{aq_{1}}{p_{1}}+\frac{dp_{1}}{q_{1}}=\frac{\pi_{12}(1-\pi_{1})}{\pi_{1}}+\frac{(1-\pi^{*})\pi_{1}}{(1-\pi_{1})}$ | $S_{Cle}=3558.1$ |
| 37 | BUB1, BUB2 | Baroni-Urabani & Buser (1976) (64) | $S_{BUB1}=\frac{a+\sqrt{ad}}{a+b+c+\sqrt{ad}}=\frac{\pi_{12}+\sqrt{\pi_{12}(1-\pi^{*})}}{\pi^{*}+\sqrt{\pi_{12}(1-\pi^{*})}}$  $S_{BUB2}=\frac{a-b-c+\sqrt{ad}}{a+b+c+\sqrt{ad}}=\frac{2\pi_{12}-\pi^{*}+\sqrt{\pi_{12}(1-\pi^{*})}}{\pi^{*}+\sqrt{\pi_{12}(1-\pi^{*})}}$ | $S_{BUB1}=0.558$  $S_{BUB2}=0.116$ |
| 38 | KF1, KF2 | Kent & Foster (1977) (65) | $S_{KF1}=\frac{-bc}{bp_{1}+cp_{2}+bc}=\frac{-({\pi_{1}-\pi}_{12})({\pi_{2}-\pi}_{12})}{({\pi_{1}-\pi}_{12})\pi_{1}+({\pi_{2}-\pi}_{12})\pi_{2}+({\pi_{1}-\pi}_{12})({\pi_{2}-\pi}_{12})}$  $S_{BUB2}=\frac{-bc}{bq_{1}+cq_{2}+bc}=\frac{-({\pi_{1}-\pi}_{12})({\pi_{2}-\pi}_{12})}{({\pi_{1}-\pi}_{12})(1-\pi_{1})+({\pi_{2}-\pi}_{12})(1-\pi_{2})+({\pi_{1}-\pi}_{12})({\pi_{2}-\pi}_{12})}$ | $S_{KF1}=-0.248$  $S_{BUB2}=-0.053$ |
| 39 | HL | Harris & Lahey (1978) (66) | $S_{HL}=\frac{a(q_{1}+q_{2})}{2(a+b+c)}+\frac{d(p_{1}+p_{2})}{2(b+c+d)}=\frac{\pi_{12}(2-\pi_{1}{-\pi}_{2})}{2(\pi^{*})}+\frac{(1-\pi^{*})({\pi_{1}+\pi}_{2})}{2({1-\pi}_{12})}$ | $S_{HL}=2441.7$ |
| 40 | Digby | Digby (1983) (67) | $S_{Digby}=\frac{\left( ad \right)^{3/4}-\left( bc \right)^{3/4}}{\left( ad \right)^{3/4}+\left( bc \right)^{3/4}}=\frac{\left( \pi_{12}(1-\pi^{*}) \right)^{3/4}-\left( ({\pi_{1}-\pi}_{12})({\pi_{2}-\pi}_{12}) \right)^{3/4}}{\left( \pi_{12}(1-\pi^{*}) \right)^{3/4}+\left( ({\pi_{1}-\pi}_{12})({\pi_{2}-\pi}_{12}) \right)^{3/4}}$ | $S_{Digby}=0.481$ |

$$where \pi^{*}=P\left( X^{*} \right)=\pi_{1}{+\pi}_{2}-\pi_{12}$$

**References**

1. Bahadur RR. A representation of the joint distribution of responses to n dichotomous items. In Studies in Item Analysis and Prediction, Vol. VI, Stanford Mathematical Studies in the Social Sciences. Stanford University Press: Stanford, CA; 1961 158‐168 p.

2. McMurray JJV, Packer M, Desai AS, et al. Angiotensin–Neprilysin Inhibition versus Enalapril in Heart Failure. *N. Engl. J. Med.* [electronic article]. 2014;371(11):993–1004. (http://www.ncbi.nlm.nih.gov/pubmed/25176015%5Cnhttp://www.nejm.org/doi/10.1056/NEJMoa1409077)

3. Warrens MJ. On Association Coefficients for 2x2 Tables and Properties That Do Not Depend on the Marginal Distributions. *Psychometrika*. 2008;73(4):777–789.

4. Warrens MJ. Similarity Coefficients for Binary Data. 2008 253 p.(https://openaccess.leidenuniv.nl/bitstream/handle/1887/12987/Full?sequence=2)

5. Warrens MJ. Chance-corrected measures for 2 × 2 tables that coincide with weighted kappa. *Br. J. Math. Stat. Psychol.* 2011;64(Pt 2):355–65.

6. Warrens MJ. On Similarity Coefficients for 2x2 Tables and Correction for Chance. *Psychometrika*. 2008;73(3):487–502.

7. Todeschini R, Consonni V, Xiang H, et al. Similarity Coefficients for Binary Chemoinformatics Data: Overview and Extended Comparison Using Simulated and Real Data Sets. *J. Chem. Inf. Model.* [electronic article]. 2012;52(11):2884–2901. (http://pubs.acs.org/doi/abs/10.1021/ci300261r). (Accessed November 16, 2016)

8. Peirce CS. The numerical measure of the success of predictions. *Science*. 1884;4(93):453–4.

9. Doolitle MH. The verification of predictions. *Bull. Philos. Soc. Washingt.* 1885;7:122–127.

10. Pearson K. On the coefficient of racial likeness. *Biometrika*. 1926;9:105–107.

11. Yule GU. On the association of attributes in statistics. *Philos. Trans. R. Soc. A*. 1900;75:257–319.

12. Montgomery AC, Crittenden KS. Improving coding reliability for open-ended questions. *Public Opin. Q.* 1977;41:235–243.

13. Yule GU. An introduction to the theory of statistics. C. Griffin and company, limited; 1932 434 p.

14. Forbes SA. On the local distribution of certain Illinois fishes: An essay in statistical ecology. *Bull. Illinois State Lab. Nat. Hist.* 1907;(7):273–303.

15. Jaccard P. The distribution of the flora in the alpine zone. *New Phytol.* 1912;11:37–50.

16. Yule G. On the methods of measuring the association between two attributes. *J. Roy. Stat. Soc.,*. 1912;75:579–652.

17. Pearson K, Heron D. On theories of association. *Biometrika*. 1913;(9):159–315.

18. Gleason HA. Some applications of the quadrat method. *Bull. Torrey Bot. Club*. 1920;47:21–33.

19. Dice L. Measures of the Amount of Ecologic Association Between Species. *Ecology*. 1945;26(3):297–302.

20. Sørensen T. A method of establishing groups of equal amplitude in plant sociology based on similarity of species and its application to analyses of the vegetation on Danish commons. *K. Danske Vidensk. Selsk.* 1948;5(4):1–34.

21. Czekanowski J. Zarys metod statystycznych w zastosowaniu do antropologii. Warszawa: 1913.

22. Nei M, Li WH. Mathematical model for studying genetic variation in terms of restriction endonucleases. *Proc. Natl. Acad. Sci. U. S. A.* 1979;76(10):5269–73.

23. Michael EL. Marine ecology and the coefficient of association. *J. Anim. Ecol.* 1920;8:54–59.

24. Kulczynski S. Die Pflanzenassoziationen der Pieninen. 1927 57-203 p.

25. Driver HE, Kroeber AL. Quantitative expression of cultural relationship. *Univ. Calif. Publ. Am. Archaeol. Ethnol.* 1932;31:211–256.

26. Braun-Blanquet J. Plant Sociology: The Study of Plant Communities. Authorized English translation of Pflanzensoziologie. McGraw-Hil. New York: 1932.

27. Ochiai A. Zoogeographical studies on the soleoid fishes found Japan and its neighboring regions. *Bull. Japan. Soc. Sci. Fish*. 1957;22(9):526–30.

28. Fowlkes EB, Mallow CL. A method for comparing two hierarchical clusterings. *J. Am. Stat. Assoc.* 1983;78(553–569).

29. Kuder GF, Richardson MW. The theory of estimation of test reliability. *Psychometrika*. 1937;2:151–160.

30. Cronbach LJ. Coefficient alpha and the internal structure of tests. *Psychometrika*. 1951;16:297–334.

31. Russell P, Rao T. On habitat and association of species of anophelinae larvae in south-eastern Madras. *J. Malar. Inst. India*. 1940;3:153–178.

32. Fuxman Bass JI, Diallo A, Nelson J, et al. Using networks to measure similarity between genes: association index selection. *Nat. Methods*. 2013;10(12):1169–76.

33. Simpson GG. Mammals and the nature of continents. *Am. J. Sci.* 1943;241:1–31.

34. Wallace DL. A method for comparing two hierarchical clusterings: Comment. *J. Am. Stat. Assoc.* 1983;78:569–576.

35. Post WJ, Snijders TAB. Nonparametric unfolding models for dichotomous data. *Methodika,*. 1993;7:130–156.

36. Loevinger JA. A systematic approach to the construction and evaluation of tests of ability. *Psychometrika*. 1947;Monograph.

37. Loevinger JA. The technique of homogeneous tests compared with some aspects of scale analysis and factor analysis. *Psychol. Bull.* 1948;45:507–530.

38. Mokken RJ. A Theory and Procedure of Scale Analysis. The Netherlands: Mouton: The Hague; 1971.

39. Sijtsma K, Molenaar IJ. Introduction to Nonparametric Item Response Theory. Thousand Oaks: 2002.

40. Cole LC. The Measurement of Interspecific Associaton. *Ecology*. 1949;30(4):411–424.

41. Goodman LA, Kruscal WH. Measures of association for cross classifications. *J. Am. Stat. Assoc.* 1954;49:732–764.

42. Scott W. Reliability of content analysis: The case of nominal scale coding. *Public Opin. Q.* 1955;

43. Sokal R, Michener C. A statistical method for evaluating systematic relationships. *Univ. Kansas Sci. Bull.* 1958;28:1409–1438.

44. Rand W. Objective criteria for the evaluation of clustering methods. *J. Am. Stat. Assoc.* 1971;66:846–850.

45. Brennan R, Light R. Measuring agreement when two observers classify people into categories not defined in advance. *Br. J. Math. …*. 1974;27:154–163.

46. Sorgenfrei T. Molluscan Assemblages From the Marine Middle Miocene of South Jutland and Their Environments. Copenhagen: Reitzel: 1958.

47. Cheetham A, Hazel J. Binary (presence-absence) similarity coefficients. *J. Paleontol.* 1969;(43):1130–1136.

48. Cohen J. A coefficient of agreement for nominal scales. *Educ. Psychol. Meas.* 1960;20:37–46.

49. Rogers DJ, Tanimoto TT. A Computer Program for Classifying Plants. *Science*. 1960;132(3434):1115–8.

50. Farkas GM. Correction for bias present in a method of calculating interobserver agreement. *J. Appl. Behav. Anal.* 1978;11:188.

51. Stiles HE. The association factor in information retrieval. *J. Assoc. Comput. Mach.* 1961;8:271–279.

52. Hamann U. Merkmalsbestand und Verwandtschaftsbeziehungen der Farinose. Ein Betrag zum System der Monokotyledonen. *Willdenowia*. 1961;2:639–768.

53. Holley JW, Guilford JP. A note on the G−index of agreement. *Educ. Psychol. Meas.* 1964;24:749–753.

54. Hubert LJ. Nominal scale response agreement as a generalized correlation. *Br. J. Math. Stat. Psychol.* 1977;30:98–103.

55. Mountford MD. An index of similarity and its applications to classificatory problems. In P. W. M. London: Butterworths: Progress in Soil Zoology; 1962 43-50 p.

56. Fager EW, McGowan JA. Zooplankton Species Groups in the North Pacific: Co-occurrences of species can be used to derive groups whose members react similarly to water-mass types. *Science*. 1963;140(3566):453–60.

57. Sokal RR, Sneath PHA. Principles of Numerical Taxonomy. San Francisco: 1963 129 p.

58. McConnaughey BH. The determination and analysis of plankton communities. *Mar. Res.* 1964;Special No:1–40.

59. Rogot E, Goldbert ID. A proposed index for measuring agreement in test-retest studies. *J. Chronic Dis.* 1966;19:991–1006.

60. Johnson SC. Hierarchical clustering schemes. *Psychometrika*. 1967;32(3):241–54.

61. Hawkins RP, Dotson VA. Reliability scores that delude: An Alice in Wonderland trip through the misleading characteristics of interobserver agreement scores in interval coding. In: *Behavior Analysis: Areas of Research and Application*. Englewood Cliffs: Prentice-Hall; 1968

62. Fleiss JL. Measuring agreement between two judges on the presence or absence of a trait. *Biometrics*. 1975;31(3):651–9.

63. Clement PW. A formula for computing inter-observer agreement. *Psychol. Rep.* 1076;39:257–258.

64. Baroni-Urbani C, Buser MW. Similarity of binary data. *Syst. Zool.* 1976;25:251–259.

65. Kent RN, Fosterl SL. Direct observational procedures: Methodological issues in naturalistic settings. In: *Handbook of Behavioral Assessment*. New York, NY: John Wiley & Sons; 1977:279–328.

66. Harris FC, Lahey BB. A method for combining occurrence and nonoccurrence interobserver agreement scores. *J. Appl. Behav. Anal.* 1978;11(4):523–7.

67. Digby PG. Approximating the tetrachoric correlation coefficient. *Biometrics*. 1983;39:753–757.
