# Supplementary material for: Bin-CE: A comprehensive web application to decide upon the best set of outcomes to be combined in a binary composite endpoint
Source: PLoS One. 2018 Dec 13;13(12):e0209000. doi: 10.1371/journal.pone.0209000 (PMC6292611; doi:10.1371/journal.pone.0209000)
Supplement: S3 File — (DOCX) [file pone.0209000.s003.docx]

**How Bin-CE runs: The mathematical functions.**

Let $X_{ijk}$ be the binary response of the *j-th* patient (j = 1, … , n_i_) in the *i-th* group (i = 1,2) for the *k-th* outcome (k = 1, …, K), where $E\left( X_{ijk} \right)=\pi_{ik}$ and $V\left( X_{ijk} \right)=\pi_{ik}(1-\pi_{ik})$. A binary CE that combines two outcomes, the RE ($X_{ijk=1}$) and the k’-th AE ($X_{ijk=k'}$), we denote this as $X_{ij}^{1k'}$ and note that is equal to 1 whenever $X_{ijk=1} or X_{ijk=k'}$ are equal to 1 and 0 otherwise, where:

$E\left( X_{ij}^{1k'} \right)=\pi_{i,1k'}^{*}=\pi_{i1}+\pi_{ik'}-\pi_{i,1k'}$ (1)

Where $\pi_{i,1k'}$ denotes the joint probability between RE and the k’-th AE outcome.

Bin-CE, in a first step computes the SSR for all possible two-components combinations of the RE (k=1) with every AE (k’=2 … K). The SSR is calculated using the appropriate sample size function which depends on the Type I and Type II errors and the type of hypotheses testing selected.

${SSR}_{1k'}=f\left( \pi_{i=1,1k'}^{*},\pi_{i=2,1k'}^{*} \right)$, for k’=2…K. (2)

The CE probability ${(\pi}_{i=1,1k'}^{*})$ of a specific combination of outcomes ($X_{ij}^{1k'})$for the **control group** can be calculated using (1)**.** However, the CE probability for the **treatment group** ($\pi_{i=2,1k'}^{*}$) must be estimated, since the probability of each outcome and the joint probability for the treatment group is unknown. Bin-CE will compute the probability of each outcome ($\pi_{i=2,k}$) and the joint probability between pairs of outcomes ($\pi_{i=2,kk'}$) for the treatment group.

The probability of each outcome in the treatment group can be estimated using the probability of the same outcome in control group and the RR for the same outcome as: $\pi_{i=2,k}=\pi_{i=1,k}*{RR}_{k}$,

Bin-CE assumes that joint probability of a pair of outcomes in the treatment group is comparable with the joint probability in control group. The estimation of the joint probability in the treatment group will be assessed depending on the direction and magnitude of the joint probability in the control group. Note that the value for the joint probability will fall in the range between the lower (*Low*) and the upper (*Up*) Fréchet bounds. Also note that the joint probability of the outcome in the treatment group must have the same direction (positive/negative) and magnitude of association (high/low) as the joint probability in the control group.

The following calculations will be performed for each pair of outcomes depending of the direction of the association:

1. *For a negative association* $\left( \pi_{kk', i=1}\leq\pi_{k,j=1}\pi_{k', i=1} \right)$

$\pi_{kk', i=2}=\frac{\pi_{kk', i=1}}{E(\pi_{kk',i=1})-Low\left( \pi_{kk',i=1} \right)}\left[ E(\pi_{kk',i=2})-Low\left( \pi_{kk',i=2} \right) \right]$, where $E\left( \pi_{kk^{'},i} \right)=\pi_{ki}\pi_{k'i}$

1. *For a positive association* $\left( \pi_{kk', i=1}\geq\pi_{k,i=1}\pi_{k', i=1} \right)$

$$\pi_{kk', i=2}=E\left( \pi_{kk',i=2} \right)+\left[ \left( Up\left( \pi_{kk',i=2} \right)-E\left( \pi_{kk',i=2} \right) \right)* \frac{\pi_{kk', i=1}-E\left( \pi_{kk',i=1} \right)}{Up\left( \pi_{kk^{'},i=1} \right)-E\left( \pi_{kk',i=1} \right)} \right]$$

Once all the treatment group parameters are estimated, the probability for each combination of two-outcomes CE in the treatment group can be determinate using (1). Note that now the effect on CE can be estimated using the Relative Risk as follows:

${RR}_{kk'}=\frac{\pi_{i=2,kk'}^{*}}{\pi_{i=1,kk'}^{*}}=\frac{\left( \pi_{i=1,k}{RR}_{k} \right)+\left( \pi_{i=1,k'}{RR}_{k'} \right)-\pi_{i=2,kk'}}{\pi_{i=1,k}+\pi_{i=1,k'}-\pi_{i=1,kk'}}$ (3)

Where $\pi_{i=2,kk'}$ will be estimated depending on the direction and magnitude of the joint probability between the outcomes k and k’ in the control group.

Once Bin-CE has estimated the SSR for all combinations of two-outcomes (the RE and each AE), it will select the CE with the lowest SSR (if any). The selected CE will be considered as the new RE for the next step and the components of this CE will be removed from the new pool of outcomes. Bin-CE will estimate the parameters for this new RE, the CE: the probability of the CE in the control group, which will be estimated using (1), the RR which will be estimated using (3) and the joint probability between the CE and the remaining AEs. The estimation of the joint probability is performed as shown in ‘*Step 2: An iterative Algorithm’* section and Figure 5 of the manuscript.

At this point, Bin-CE has reduced a k-dimensional problem to a k-1-dimensional and it will continue the iterative process until there are no outcomes left or until there are no new combinations which reduce the SSR.
